# Indonesian optimal sarcopenia cutoff values of calf circumference, muscle strength, and physical performance: a multicenter descriptive and cross-sectional study

**DOI:** 10.3389/fmed.2025.1536848

**Published:** 2025-07-17

**Authors:** Siti Setiati, Shahrul Bahyah Kamaruzzaman, Samuel Teong Huang Chew, Nina Kemala Sari, Kuntjoro Harimurti, Purwita Wijaya Laksmi, I Gusti Putu Suka Aryana, Sri Sunarti, Noto Dwimartutie, Ika Fitriana, Anastasia Asylia Dinakrisma, Muhammad Khifzhon Azwar, Roza Mulyana, Dina Aprillia Ariestine, Wiwit Agung Sri Nur Cahyawati, Lazuardhi Dwipa, Fatichati Budiningsih, Agus Sudarso, Mala Hayati, Chacha Marisa Isfandiari, Muhammad Darma Muda Setia, Rahmi Istanti

**Affiliations:** ^1^Division of Geriatrics, Department of Internal Medicine, Cipto Mangunkusumo National General Hospital – Faculty of Medicine Universitas Indonesia, Jakarta, Indonesia; ^2^Division of Geriatric Medicine, Department of Medicine, Faculty of Medicine, University of Malaya, Kuala Lumpur, Malaysia; ^3^Ageing and Age-Associated Disorders Research Group, Faculty of Medicine, University of Malaya, Kuala Lumpur, Malaysia; ^4^Department of Geriatric Medicine, Changi General Hospital, Singapore, Singapore; ^5^SingHealth Duke-NUS Medicine Academic Clinical Programme, Singapore, Singapore; ^6^Yong Loo Lin School of Medicine, National University of Singapore, Singapore, Singapore; ^7^Division of Geriatrics, Department of Internal Medicine, Faculty of Medicine, Universitas Udayana – Prof. Dr. Ngoerah General Hospital Denpasar, Denpasar, Indonesia; ^8^Geriatric and Medical Gerontology Division, Internal Medicine Department, Medical Faculty of Brawijaya University, Malang, Indonesia; ^9^Department of Internal Medicine, Faculty of Medicine, Universitas Andalas – Dr. M. Djamil General Hospital, Padang, Indonesia; ^10^Department of Internal Medicine, Faculty of Medicine, Universitas Sumatera Utara, Medan, Indonesia; ^11^Prof. Chairuddin P. Lubis Universitas Sumatera Utara Hospital, Medan, Indonesia; ^12^Division of Geriatrics, Department of Internal Medicine, Ulin District General Hospital, Banjarmasin, Indonesia; ^13^Department of Internal Medicine, Moch Ansari Saleh District General Hospital, Banjarmasin, Indonesia; ^14^Division of Geriatrics, Department of Internal Medicine, Faculty of Medicine, Universitas Padjadjaran – Hasan Sadikin General Hospital, Bandung, Indonesia; ^15^Division of Geriatrics, Department of Internal Medicine, Faculty of Medicine, Universitas Sebelas Maret – Dr Moewardi Hospital, Surakarta, Indonesia; ^16^Division of Geriatrics, Department of Internal Medicine, Faculty of Medicine, Universitas Hasanuddin – Wahidin Sudirohusodo General Hospital, Makassar, Indonesia; ^17^Merauke District General Hospital, Merauke, Indonesia; ^18^Division of Geriatrics, Department of Internal Medicine, Faculty of Medicine, Universitas Syiah Kuala, Banda Aceh, Indonesia; ^19^Clinical Epidemiology and Evidence Based Medicine Unit, Cipto Mangunkusumo Hospital – Faculty of Medicine Universitas Indonesia, Central Jakarta, Indonesia

**Keywords:** sarcopenia, walking speed, physical functional performance, muscle strength, Indonesia

## Abstract

**Introduction:**

The prevalence of possible sarcopenia among Indonesian older adults using the Asian Working Group for Sarcopenia (AWGS) 2019 consensus cutoffs was abnormally high compared with reported prevalence in other East Asian countries. This suggests that the current AWGS cutoff values may not be applicable to the Indonesian population as it did not contain reference statistical values based on the local population normative studies. This lack of local data may result in inaccurate assessments and diagnoses of sarcopenia among Indonesian population. We therefore aimed to conduct a nationwide study in the community to obtain optimal cutoff values for calf circumference, muscle strength, and physical performance for the diagnosis of sarcopenia in Indonesia.

**Methods:**

In this multicenter descriptive and cross-sectional study, we collected data from healthy adults aged 20–39 years in different islands in Indonesia. We followed the recommendation by the European Working Group for Sarcopenia in Older People (EWGSOP) of using 2 standard deviations [SD] below the mean of a young healthy cohort for the new cutoff points of sex-specific handgrip strength, calf circumference, and gait speed. The mean +2 SD was used for five times sit-to-stand test (FTSTST).

**Results:**

The mean −2 SD value of male handgrip strength was 21.15 kg, and 14.34 kg for female subjects. The mean −2 SD value of male and female calf circumferences were 29.92 cm, and 26.70 cm, respectively. Two SD below the mean of gait speed results was 0.51 s, whereas 2 SD above mean of FTSTST results was 11.69 s.

**Discussion:**

The suggested cutoff values for low calf circumference, muscle strength, and gait speed in Indonesian population are different from those in previous consensuses. Body morphology, culture, and obesity may be the factors related to this phenomenon. This study contributes to the body of literature of normative data used to determine cutoff values used in the diagnosis of sarcopenia in Indonesian and Asian populations.

## 1 Introduction

The Asian Working Group for Sarcopenia (AWGS) pioneered the development of diagnostic criteria specific to the Asian population, considering factors such as body composition, muscle strength, and physical performance (1). Systematic review and meta-analysis of the studies following AWGS 2014 guideline concluded that the prevalence of sarcopenia in Asian older adults was as high as 14% (95% confidence interval [CI] 0.12–0.17) (2). Prevalence of sarcopenia in Asian populations is comparable to that of Caucasian populations when using appropriate diagnostic criteria (3). The AWGS 2014 guideline addressed the cutoff points for older Asian people by modifying the European Working Group on Sarcopenia in Older People (EWGSOP) guideline (1), followed by publishing updated guideline in 2019 (4). AWGS 2019 diagnostic algorithm incorporated calf circumference for muscle mass assessment, handgrip strength to assess muscle strength, five times sit-to-stand test (FTSTST), and gait speed to assess physical performance (4). Various studies have highlighted physiological and health-related factors which may affect physical performance, such as muscle strength, age, pain, and cognitive function (5–7). There is also a growing recognition of the influence of ethnicity, cultural background, and even geolocation differences of the same ethnicity on the various components of body composition and muscle health (8).

The most populous nations in Southeast and East Asian regions, namely, Indonesian and People’s Republic of China, were not represented among the study populations and were used to determine the cutoffs in both versions of the AWGS consensus guidelines (1, 4). FTSTST was used as a surrogate for gait speed in a single-center Japanese study, with the cutoff for FTSTST estimated based on the linear regression study between FTSTST and gait speed by Nishimura et al. (9). While China had solved the problem by conducting a study to determine the optimal cutoff points for the diagnosis of sarcopenia among mainland Chinese population (10), there was a lack of Indonesian data in AWGS cutoff points. Our previous SARC-F questionnaire-based multicentre study found that 17.6% of Indonesian older adults were at risk of sarcopenia (10). However, a cross-sectional study in Indonesia by Sumandar et al. based on AWGS 2019 cutoff points (4) reported an abnormally high prevalence of possible sarcopenia (45.5%) among Indonesian older adults (11). Sumandar, et al. suggested that the use of current AWGS 2019 cutoff values for the diagnosis of sarcopenia may result in an overestimation of the prevalence of sarcopenia in Indonesia (11).

The cutoffs for the various components of muscle health used in the diagnosis of sarcopenia in the AWGS 2019 are based on a combination of studies using 2-standard deviations, 1-standard deviation, or the lowest quintiles, a cutoff for normality (1, 4). On the other hand, the consensus form the European Working Group on Sarcopenia in Older People in 2010 (EWGSOP) relied on 2 standard deviations (SD) below mean of the numerical data obtained from healthy young adult population as reference statistical value to classify abnormal findings (12, 13).

The abnormally high prevalence of possible sarcopenia reported by Sumandar et al. using cutoff points from AWGS 2019, together with our concerns regarding the lack of Indonesian normative data, and the use of variable standard deviations and lowest quintiles as reference statistical values were the impetus for us to conduct this descriptive and cross-sectional study in an Indonesian healthy young adult population. We aimed to conduct a multicentre study to obtain Indonesia’s optimal sarcopenia cutoff values for calf circumference, muscle strength, FTSTST, and gait speed. We also aimed to use the 2-standard deviations based on the mean of a healthy young adult population as the cutoff for normality as per the European Working Group on Sarcopenia in Older People in 2019 (EWGSOP2) guidelines (12, 13), with slight method adjustment for FTSTST.

## 2 Materials and methods

### 2.1 Study design and subjects

We conducted a multicenter descriptive and cross-sectional study in multiple study centers. We utilized consecutive sampling method to collect the data from healthy Indonesian adults aged 20–39 years who met predetermined criteria. The inclusion criterion was the ability to walk without walking aid (e.g., walking stick, frame, wheeled trolley, crutches, walkers, or rollators). The exclusion criteria were history of electronic device or orthopedic metal implantations, heart failure, chronic kidney disease, peripheral oedema, diuretic consumption, hyperthyroidism or hypothyroidism, and later stage of malignancies with cachexia. The history was ascertained through history taking. The hospitals involved in data collection process were Dr. Cipto Mangunkusumo National General Hospital, Jakarta; Prof. Dr. I Goesti Ngoerah Gde Ngoerah Central General Hospital, Denpasar; Dr. Hasan Sadikin Central General Hospital, Bandung; Dr. Zainoel Abidin District General Hospital, Banda Aceh; Dr. M. Djamil Central General Hospital, Padang; Dr. Wahidin Sudirohusodo Central General Hospital, Makassar; Dr. Moewardi District General Hospital, Surakarta; Dr. Saiful Anwar District General Hospital, Malang; Dr. H. Moch. Ansari District General Hospital, Banjarmasin; Prof. Chairuddin P. Lubis Hospital, Medan; and Merauke District General Hospital, Merauke.

We calculated the minimum sample sizes utilizing sensitivity analysis formula. The highest number of calculation result was considered the minimum sample size, which was 676, consisting of 376 of male subjects and 300 of female subjects. We determined the minimum sample size for each of 6 main islands (Java, Bali, Kalimantan [Borneo], Papua, Sumatra, and Sulawesi [Celebes]) based on the ratio of the regional population to the total population in 2022 (*n* = 252,021,000). Therefore, the minimum sample size calculated for Java was 388 subjects (216 male subjects and 172 female subjects), whereas it was 12 for Bali (7 male subjects and 5 female subjects) and 46 for Kalimantan (25 male subjects and 20 female subjects). The minimum sample size was 15 for Papua (8 male subjects and 7 female subjects), 161 for Sumatra (89 male subjects and 71 female subjects), and 54 for Sulawesi (30 male subjects and 24 female subjects). The minimum sample size for Java was divided into four centers, namely, Jakarta, Bandung, Surakarta, and Malang. Centers in Banda Aceh, Medan, and Padang represented the population of Sumatra, and the minimum sample size for this island was divided into three centers. Makassar represented the population of Sulawesi (Celebes). On the other hand, centers in Denpasar, Banjarmasin, and Merauke represented the population of Bali, Kalimantan (Borneo), and Papua, respectively.

Ethics approval was obtained from the Ethics Committee of the Faculty of Medicine, Universitas Indonesia–Cipto Mangunkusumo Hospital, Jakarta, Indonesia, with registration number KET-712/UN2.F1/ETIK/PPM.00.02/2023. Each subject provided their written informed consent to participate in this study.

### 2.2 Data collection

Calf circumference was measured in centimeter (cm) at the point where the calf appeared to be the largest. Muscle strength was assessed by handgrip strength assessment. Handgrip strength was documented in kilogram (kg) using hydraulic dynamometer (Jamar Hydraulic Hand Dynamometer, Model J00105, Lafayette Instrument, Lafayette, Indiana, United States). We defined handgrip strength as the highest grip strength of the dominant handgrip of three attempts with maximum effort of isometric contractions. Test was done in sitting position with 90-degree elbow flexion following the recommended standard (2).

We assessed the physical performance through FTSTST and gait speed assessments. The former was done prior to the latter. To assess FTSTST, we asked the study subjects to sit down on a chair with back support initially. The subjects were asked to move from sitting position to standing position five times in a row non-stop as quick as possible. The measurement stopped when the individual stands for the fifth time. For gait speed assessment, we instructed each study subject to walk on their usual walking pace for 6 m without deceleration. The patient was not forced to walk as fast as possible. Walking attempts were made two times, and the average of both results was considered as the subject’s gait speed.

### 2.3 Statistical analyses

Statistical analyses relied on SPSS version 21 (IBM, Armonk, New York, United States). We provided the characteristics of study subjects descriptively. Numerical data with normal distribution were reported as mean (standard deviation [SD]). We reported numerical data with skewed data distribution by providing the median (interquartile range [IQR]). We determined cutoff points for sex-specific handgrip strength and calf circumference, as well as for general gait speed, by finding 2 SD below the mean of each variable (13, 14). Values less than the 2 SD below mean for gait speed were classified as low physical performance. Values less than the 2 SD below mean for sex-specific handgrip strength and calf circumference were considered low or abnormal. We also showed the SD value above mean and 20th percentile for FTSTST. We also provided the 20th percentile value of each variable, as an alternative method used previously by sarcopenia experts in China (14).

## 3 Results

We collected data from 905 Indonesian adults (Table 1). The mean age of the subjects was 26.98 years. Of all study subjects, 22.7% of study subjects were from Jakarta and 55.9% were female. A larger proportion of study subjects were either overweight or obese (53.3%). Each study center exceeded the minimum sample size required in this study.

**Table 1:** Characteristics of study population (*n* = 905).

Characteristics, unit if indicated	Frequency (*n*)	Percentage (%)
Age, years, mean (SD)	26.98 (4.91)	
Sex		
Male	399	44.1
Female	506	55.9
Body weight, kg, median (IQR)	65.41 (15.31)	
Body height, cm, mean (SD)	162.67 (8.25)	
Body mass index, kg/m^2^, median (IQR)	23.83 (5.46)	
Nutritional status		
Underweight (<18.5 kg/m^2^)	51	5.6
Normal body mass index (18.5–23.5 kg/m^2^)	372	41.1
Overweight and obesity (>23.5 kg/m^2^)	482	53.3
Study center		
Aceh	54	5.9
Bandung	91	10
Banjarmasin	78	8.6
Denpasar	15	1.7
Jakarta	206	22.7
Makassar	53	5.9
Malang	95	10.5
Medan	54	5.9
Merauke	109	12.0
Padang	56	6.2
Surakarta	94	10.4
Last completed formal education		
No formal education	5	0.6
Primary school	0	0
Junior high school	4	0.4
Senior high school	109	12
Higher education	787	86.9

The data were reported in frequency and percentage, unless stated otherwise.

IQR, interquartile range; SARC-CalF, SARC-F combined with calf circumference; SD, standard deviation; SPPB, The Short Physical Performance Battery.

The mean −2 SD value of male subject handgrip strength was 21.15 kg, and 14.34 kg for female subjects. The mean −2 SD value of male subject calf circumference was 29.92 cm, and 26.70 cm for female subjects. The mean +2 SD value of FTSTST result of all subjects was 11.69 s. The mean −2 SD value of gait speed of all subjects was 0.51 m/s (Table 2).

**Table 2:** Mean, standard deviation, 2 standard deviations below mean, median, and 20th percentile of the data from different study variables.

Diagnostic component (unit)	Results
Male handgrip strength (kg)	
Mean	38.95
SD	8.90
Mean −2 SD	21.15
Median	38
20th percentile	31
Female handgrip strength (kg)	
Mean	25.45
SD	5.56
Mean −2 SD	14.34
Median	25
20th percentile	20.30
Male calf circumference (cm)	
Mean	38.13
SD	4.11
Mean −2 SD	29.92
Median	38
20th percentile	35.00
Female calf circumference (cm)	
Mean	34.74
SD	4.02
Mean −2 SD	26.70
Median	34
20th percentile	31.50
Five times sit-to-stand test (s)	
Mean	7.43
SD	2.13
Mean +2 SD	11.69
Median	7.07
20th percentile	5.55
Gait speed (m/s)	
Mean	1.09
SD	0.29
Mean −2 SD	0.51
Median	1.09
20th percentile	0.87

Two standard deviations above mean were used for five times sit-to-stand test.

SD, standard deviation.

## 4 Discussion

These suggested cutoff points are different from the criteria set by AWGS in 2019. To detect low calf circumference, we suggested using <29.92 cm and <26.70 cm for male and female Indonesians, respectively. The values are much lower than the reference values in AWGS consensus (<34 cm for male subjects and <33 cm for female subjects) (4). Similarly, the suggested cutoff points to detect lower handgrip strength for male and female Indonesians are also lower than the values in AWGS consensus (<21.15 kg vs. < 28 kg for male subjects; and <14.34 kg vs. < 18 kg for female subjects) (4). Handgrip strength has been identified as a reliable proxy for muscle mass in older Asian adults (15), and therefore, appropriate cutoff point is crucial for handgrip strength assessment.

Low physical performance may be detected in Indonesians walking at pace slower than 0.51 m/s. The cutoff pace is much lower than the values in AWGS and EWGSOP2 consensuses (4, 14). Despite the different methods used in determining the optimal FTSTST cutoff value between our study and the study conducted by Nishimura et al. (9), our proposed cutoff point of FTSTST (11.69 s) is similar to that of AWGS consensus (12 s) (4). FTSTST alone was not included in EWGSOP2’s sarcopenia diagnostic algorithm, although it was incorporated in the short physical performance battery (SPPB) (12, 13).

There are several possible reasons why muscle-related assessment result in healthy Indonesian adults may not be comparable to that of other Asian populations. First, lower mean result and cutoff points of sarcopenia assessment of the lower limb may be related to being overweight and obesity. More than half of our study subjects were either overweight or obese (53.3%). The proportion of overweight and obese young adults in our study was similar to the most recent Indonesian Health Survey result in 2023. Being overweight and obese were found in 26.2% of Indonesians aged 20–24 years, 41.9% of those aged 25–29 years, 50.9% of those aged 30–34 years, and 55.4% of those aged 35–39 years (16). In contrast, the prevalence of obesity among Singaporeans aged 30–39 years was only 12.4% (17). Reduced lower limb strength relative to body mass in overweight and obese individuals may be affected by a reduced functional capacity (e.g., walking difficulties, stairs negotiation, and rising from a chair or bed) (18, 19), neural adaptations, and changes in muscle morphology (19).

Second, developed Asian nations, from which the AWGS data were obtained, may have different body morphologies compared with the body morphology of Indonesians. Body growth is multifactorial (20), and this might indirectly affect an individual’s physical performance (e.g., gait speed). The factors included chronic/non-communicable diseases, nutrition, urbanization, socioeconomic status, physical activity, psychosocial deprivation, and climate (20). Higher income in developed Asian nations may be associated with better education, leading to improved childcare, nutrition, and better access to medical services. The improvement in different fields will increase overall stature (21, 22). One of the body dimensions was body height, which was strongly associated with gait speed, as suggested by previous study in Singapore (23). Mean (SD) of body height of subjects in our study was only 162.67 cm (8.25 cm). A comparative anthropometric study was conducted comparing Indonesian versus Singaporean population as an example of a developed Asian nation. Although both nations are technically Asian, the study showed that both female and male subjects in Singapore tend to have larger physical dimensions than Indonesians (22).

Third, it is hypothesized that local culture may affect individual’s physical performance. While various studies have highlighted physiological and health-related factors affecting physical performance, there is a growing recognition of the influence of cultural backgrounds on this aspect of mobility (5–7). Indonesian citizens only walked 3,513 steps per day and were at the 46th place of 46 countries assessed in previous study about daily step count (23). Furthermore, the mean (SD) of Indonesian healthy adult’s gait speed in our study was 1.09 m/s (±0.29 m/s), as opposed to the result in previous study among Japanese adults being 1.30 m/s (±0.10 m/s) (24). Indonesian gait characteristics include a shorter stride length of 1.15 m and a slower cadence of 110 steps per minute compared to other populations (25). Reluctance to walk was seen in a study of the population of Kupang, people of which were willing to walk an average distance of 115 m only to access public transit (26). Such habit of slow walking and reluctance to walk may be influenced by environmental factors related to safety and comfort of pedestrians in Indonesia (27). However, we should not normalize slow gait speed, as slow gait speed is a significant risk factor for all-cause and cardiovascular mortality, independent of other major risk factors (28). Slow gait speed has also been associated with a higher risk of adverse health outcomes, including all-cause mortality in adults with knee osteoarthritis (29).

This study has several strengths. First, this is the first multicentre study to estimate the sarcopenia-related measurement data of this multiethnic archipelagic state. We also determined the minimum sample size appropriately by considering regional population of each main island. Second, we followed the recommendations by the European Working Group for Sarcopenia in Older People of using 2 SD below the mean of a young healthy cohort to determine the cutoffs for normality (12, 13). Our results may also add to the body of literature with regard to the normative values for muscle health and diagnosis of sarcopenia in this region. However, we also acknowledged our limitation. This study population did not include the rural population of each island, although more than 50% of Indonesian older adults are now living in the cities (30). Caution should be exercised if extrapolating these cutoff points to other populations, as there may be distinct differences in cutoffs of other Asian groups. Future studies are required to establish optimal cutoff points in Indonesian population regarding muscle mass using other modalities of muscle mass assessment such as dual energy X-ray absorptiometry or bioelectrical impedance analysis. Third, we did not perform stratified analysis of data from this age group.

In conclusion, we propose the use of calf circumference <29.92 cm and <26.70 cm for the detection of low calf circumference among male and female Indonesians, respectively. We also propose that the optimal cutoff points to detect low handgrip strength are <21.15 kg and <14.34 kg for male and female Indonesians, respectively. Low gait speed can be defined as a walking pace <0.51 m/s. Abnormal physical performance is considered in those completing FTSTST >11.69 s. This study provides important contribution to the body of literature with regard to normative data required to determine cutoff values used in the diagnosis of sarcopenia in Indonesian and Asian populations. We suggest that the process of determining cutoff points should consider our multicentre study results. Future clinical screening or community-based programs in this region are subject to further discussion with health policymaker.

## Data Availability

The raw data supporting the conclusions of this article will be made available by the authors, without undue reservation.
